# Cell and Cell-Free Therapies to Counteract Human Premature and Physiological Aging: MSCs Come to Light

**DOI:** 10.3390/jpm11101043

**Published:** 2021-10-18

**Authors:** Arantza Infante, Clara I. Rodríguez

**Affiliations:** Stem Cells and Cell Therapy Laboratory, Biocruces Bizkaia Health Research Institute, Cruces University Hospital, 48903 Barakaldo, Spain; arantza.infantemartinez@osakidetza.eus

**Keywords:** MSCs, cell therapies, cell-free therapies, extracellular vesicles, physiological aging, premature aging, paracrine mechanism, inflammation, stem cell exhaustion

## Abstract

The progressive loss of the regenerative potential of tissues is one of the most obvious consequences of aging, driven by altered intercellular communication, cell senescence and niche-specific stem cell exhaustion, among other drivers. Mesenchymal tissues, such as bone, cartilage and fat, which originate from mesenchymal stem cell (MSC) differentiation, are especially affected by aging. Senescent MSCs show limited proliferative capacity and impairment in key defining features: their multipotent differentiation and secretory abilities, leading to diminished function and deleterious consequences for tissue homeostasis. In the past few years, several interventions to improve human healthspan by counteracting the cellular and molecular consequences of aging have moved closer to the clinic. Taking into account the MSC exhaustion occurring in aging, advanced therapies based on the potential use of young allogeneic MSCs and derivatives, such as extracellular vesicles (EVs), are gaining attention. Based on encouraging pre-clinical and clinical data, this review assesses the strong potential of MSC-based (cell and cell-free) therapies to counteract age-related consequences in both physiological and premature aging scenarios. We also discuss the mechanisms of action of these therapies and the possibility of enhancing their clinical potential by exposing MSCs to niche-relevant signals.

## 1. Introduction

Aging, an inexorable consequence of life, can be defined by a time-dependent loss of cellular and molecular homeostasis, leading to impaired function of tissues and organs that are unable to activate proper compensatory mechanisms to restore the lost functionality [1]. Affecting nearly all tissues, organs and systems in an organism, aging itself is the stronger risk for the development of age-related pathologies [2]. The consequences of aging are especially visible in tissues of mesenchymal origin, such as connective tissues (bone, cartilage and fat) and the cardiovascular system. Thus, musculoskeletal disorders, such as osteoporosis and osteoarthritis and body fat redistribution and loss, in addition to cardiovascular pathologies, are among the most common pathological features shared by the elderly, instigating chronic disability, and in consequence, long-term healthcare needs [3].

Mesenchymal stem cells (MSCs) are adult stem cells with the abilities of self-renewal and differentiation to more specialized mesenchymal lineages such as osteoblasts, chondrocytes and adipocytes [4]. Senescent MSCs accumulate in vivo gradually with aging, and in vitro, upon prolonged culture expansion. Among the main characteristics of senescent MSCs are halted proliferation and migration, impaired differentiation and the acquisition of the senescence associated secretory phenotype (SASP), which is mainly composed of proinflammatory cytokines, matrix remodeling enzymes, reactive oxygen species (ROS) and chemotactic molecules [5]. SASP is responsible for a local proinflammatory microenvironment that affects the behavior of neighboring cells, inducing their senescence, via autocrine/paracrine mechanisms [6,7,8]. In vivo, the dysfunctional status of senescent MSCs leads to “stem cell exhaustion”, a hallmark of aging, which negatively impacts on tissue homeostasis and the regeneration capacity of the organism in response to injury [1].

The potential use of MSCs as a therapy has been under extensive investigation over the last two decades, showing encouraging results in certain pathologies, including age-related ones, by restoring tissue functionality. Damaged tissue regeneration, immunomodulation and secretion of paracrine mediators are the three pillars of MSC therapeutic mechanism of action [9]. Although MSCs can migrate to the injured tissue and differentiate into specific cell types, their main therapeutic effect is thought to be paracrine, by secreting bioactive factors depending on the microenvironment they face, known as a hit-and-run mechanism [4,9,10]. Along these lines, the first studies using MSCs as a therapy in pediatric patients affected by the rare bone disorder, osteogenesis imperfecta, showed that after in vivo administration, the engraftment of MSCs in target tissues such as bone was anecdotal, although beneficial effects were found [11]. A growing body of evidence points to the secretory properties of MSCs as the underlying mechanisms behind the beneficial effects of MSCs-based therapies, rather than the initial cell replacement that MSCs were supposed to exert [11,12,13]. This assumption is supported by the fact that the active components secreted by MSCs, mainly exosomes, are themselves demonstrating efficacy as cells to treat certain pathologies [14].

The considerable increase in life expectancy over the past century is an undeniable observation [15]. However, this gained “extra” time lived at the end of people’s lives, in contrast to what is desirable, escalates the years that individuals suffer from the so-called age-related diseases. Thence, therapeutic interventions for aging are likely more helpful, from both health and economic points of view, than eradicating individual diseases, basically because with this approach, multiple diseases will be targeted [2,16]. Thus, treatments to target aging are particularly valuable, and constitute a goal that is under intensive research nowadays.

In this review, we highlight that aged MSCs, encompassing either in vivo-aged cells, coming from elderly donors, or in vitro-aged cells, derived from late culture passages, show a senescent phenotype with a decline in quality, this being key in the development of age-related pathologies. Thereby, we take advantage of the current knowledge about the mechanisms driving physiological aging and premature aging syndromes, such as those affecting the nuclear lamina (a meshwork of proteins lining the inner nuclear membrane), and the role that dysfunctional MSCs play in the pathophysiology of these diseases. We also summarize the outcomes from preclinical and clinical studies using MSC-based cell and cell-free (with extracellular vesicles (EVs) isolated from MSCs) therapies to counteract age-related diseases. Moreover, we point to the superior therapeutic potential of allogeneic young MSCs to counteract age-related diseases in addition to the enhanced potential observed in “primed” MSCs. All in all, we shed light on the coming advanced therapeutic avenues to prevent age-related pathologies and extend the healthspan, that is, the disease-free period, in a human lifetime.

## 2. Progeroid Laminopathies: A Model to Study the Relevance of MSCs in Aging

Progeroid laminopathies are very rare devastating genetic diseases characterized by early onset signs of physiological aging, hence bringing to affected patients a significant reduced lifespan. The major clinical features entail growth impairment, lipodystrophy and dermal and musculoskeletal abnormalities, in addition to cardiovascular disease, which is usually the cause of death [17]. At the cellular level, laminopathies are characterized by alterations in the nuclear lamina, a mesh of intermediate filaments—A- and B-type lamins—that form a mesh lining the inner surface of the nuclear envelope [18]. Progeroid laminopathies originate by way of mutations in genes coding for A-type lamins (*LMNA*), their binding proteins (*BANF1, LEMD2*) or in proteins that process A-type lamins (*ZMPSTE24*) [19].

Alternative splicing of *LMNA* leads to the expression of the two predominant isoforms of A-type lamins: prelamin A (the precursor of mature lamin A) and lamin C [18]. Prelamin A undergoes a series of posttranslational modifications at its C terminal region to give rise to mature lamin A. Most of progeroid laminopathies are due to mutations that are within this C-terminus, encompassing the cleavage sites for ZMPSTE24 protein, leading to the toxic accumulation at the nuclear envelope of the lamin A precursor, prelamin A, or progerin, a truncated form of lamin A. In fact, the accumulation of progerin at the nuclear envelope is the molecular hallmark of Hutchinson–Gilford Progeria Syndrome (HGPS), an extreme progeroid laminopathy. Patients show premature functional alterations in many tissues and organs of mesenchymal origin, as occurs in physiological aging. Clinical symptoms appear within the first 18 months after birth, these being growth retardation, facial dysmorphism and lipodystrophy, along with skin and musculoskeletal disorders as their cardinal features. Death occurs when affected patients are in their early teenage years (median age of 14.5 years), mainly due to cardiovascular complications [20]. There is no cure for this dramatic syndrome, although intense basic and translational research, especially supported by the Progeria Research Foundation, are ongoing. These huge efforts have recently yielded encouraging results: lonafarnib, the only drug available, recently approved by the U.S. Food and Drug Administration to treat HGPS, extends patients’ lifespan by 2.5 years [21].

Strikingly, and as occurs in physiological aging, many of the affected tissues in these *LMNA*-linked premature aging syndromes, arise from mesenchymal lineages (lipodystrophy, bone fragility, atherosclerosis). Progeroid mice models pathologically accumulating either progerin or prelamin A recapitulate the phenotypes of premature aging as well as the mesenchymal tissue affectation that patients show [22,23]. These observations suggest that the cell-type specific pathologies in aging could be due, in part, to dysfunctional MSCs. This fact is also confirmed by naturally aging MSCs, which, driven by the alteration of different molecules (such as transcription factors and miRNAs) and processes (such as autophagy), show a trend towards adipogenic differentiation at the expenses of osteogenesis [24].

The study of the consequences of the accumulation of immature forms of lamin A in human MSCs has been attempted by different in vitro approaches; first, by overexpression of progerin or prelamin A (induced genetically or as adverse effect of pharmacological treatments which inhibit the activity of ZMPSTE24), and later by the generation of induced pluripotent stem cells (iPSCs) from cells isolated from affected patients in a more disease-relevant in vitro scenario [25,26,27,28,29]. Both approaches have been key in demonstrating that abnormal accumulations of prelamin A and progerin trigger senescence in MSCs [8,26,27]. Moreover, the pathological accumulation of prelamin A leads to the sequestration at the nuclear envelope of specific transcription factors, key for adipogenic differentiation, autophagy induction and antioxidative response in MSCs such as SP1 and OCT1, a mechanism driving, at least in part, the premature aging phenotypes in these cells [26,27]. Thus, although MSCs accumulating prelamin A respond to early adipogenic differentiation cues, the aberrant prelamin A accumulation in adipocytes leads to a reduction not only in their lipid storage capacity, but also causes mitochondrial dysfunction, endoplasmic reticulum stress and an altered, age-related lipid metabolism [26,30]. Later studies performed with human MSCs derived from progeria-iPSCs confirmed this inhibitory role of the abnormal accumulation of prelamin A/progerin in the late-stage process of adipogenesis [31]. Regarding osteogenic differentiation, the accumulation of progerin has been described as impairing osteogenic differentiation from MSCs derived from progeria-iPSCs. In this circumstance, a first enhancement of the early stages of osteogenesis differentiation has been described [32,33,34,35], followed by a reduction in the mineralization capacity of these cells in later stages of osteogenesis [34].

Not only is the differentiation capacity of MSCs accumulating prelamin or progerin affected, but alterations in autophagy as well as the induction of SASP have also been described [8,27,28]. Moreover, MSCs aberrantly accumulating progerin or prelamin A show less viability, especially under stress conditions, and therefore show a diminished therapeutic potential when transplanted in a murine ischemic hind limb model [27,29].

Strikingly, the later finding of detectable amounts of prelamin A and progerin exclusively in aged human cells (fibroblasts and vascular smooth cells isolated from aged donors) and in MSCs aged in vitro, suggested a common, nuclear lamina-dependent mechanism, driving cellular aging, and reinforces the concept of age-related MSC exhaustion as a mechanism of natural aging [36,37,38,39,40].

## 3. MSCs as a Therapy for Aging

In the past few years, there have been important advances in developing strategies aimed at treat aging consequences in animal models by directly targeting senescent cells or by transferring young factors to old mice. In fact, some detrimental consequences of aging have been shown to be, to a certain extent, recoverable by these approaches. On one hand, the direct clearance of the senescent cells has become a real possibility by the use of senostatic or senolytic compounds, which specifically inhibit the SASP of senescent cells, or directly kill them. Thus, when tested in mice models of aging, these drugs have demonstrated being able to improve the healthspan and thereby ameliorate a wide range of age-associated pathologies [41,42,43]. Along these lines, the in vitro treatment of aged human MSCs with senolytics has been recently shown to selectively target a certain subset of MSCs (20–30% of total cells), which leads to the increased proliferation and osteogenic potential of the surviving ones [44]. These findings open new avenues of treatments based on senolytics, not only to eliminate senescent cells, but also to improve the functions of the remaining ones, among which are MSCs, in elderly populations [44]. In fact, the in vivo effectiveness of senolytics in humans are currently under extensive clinical research. Preliminary results from a clinical trial treating elderly individuals affected by kidney disease with senolytics show a decrease in tissue-specific senescent cells, thus confirming the mechanism of action of these drugs in vivo [45]. Moreover, the first clinical trial in humans, a pilot study testing the feasibility and potential impact of senolytics treatment in idiopathic pulmonary fibrosis, an age-related disease, reported physical improvements in patients [46].

On the other hand, young blood factors, administered directly or by heterochronic parabiosis experiments, have been able to restore the age-related decline of certain tissues and organs, and vice versa, establishing the concept of the existence of a systemic regulation of aging [47,48,49,50]. Moreover, a recent study reported that even the benefits coming from external interventions in elderly mice, such as exercise, which is considered to be a geroprotector, can be transferred to sedentary aged animals through the administration of blood components [51].

It is therefore not surprising that aging factors from blood serum have been shown to negatively impact on MSCs. Thus, heterochronic experiments culturing young MSCs in the presence of sera isolated from middle-age mice induced a cell cycle arrest and an increase in the expression of senescence markers and SASP, pointing to a direct effect of systemic aging factors on MSCs [52].

Considering this evidence, it seems reasonable to propose young MSCs or their secreted factors (cell-free therapies) as another strategy to counteract aging, where their beneficial regenerative and paracrine effects should be noticeable in tissues and organs of mesenchymal origin—those especially affected by age. Moreover, low-grade inflammation, known as inflammaging, is a feature of physiological aging [53]. Elderly individuals, progeroid mice models, as well as human HGPS cells show increased expression of the proinflammatory cytokine interleukin-6 (IL-6). Interestingly, recent preclinical evidence has shown improvements to aging features in progeroid mice treated with tocilizumab, a neutralizing antibody raised against IL-6 [54]. Thus, beyond tissue regeneration, the immunomodulatory and anti-inflammatory properties of MSCs could be undoubtedly quite appealing for counteracting this age-related inflammation.

### 3.1. MSCs Therapies to Counteract Physiological Aging

The aim of using MSCs to treat physiological aging is to recover the functionality of specific tissues or organs affected by age; for instance, knee dysfunctionality and heart failure, or to recover from frailty, a holistic concept defined as the cumulative age-related deterioration of physiological systems that show reduced capacity to the face of environmental stressors. The indicators for frailty involve exhaustion, weight loss, weak grip strength, slow walking speed and low energy expenditure and cognitive status, all characteristic of elderly individuals [3].

#### 3.1.1. Knee Osteoarthritis

Osteoarthritis (OA), a common chronic disease in older adults, is becoming a remarkable health and socio-economic burden due to its increased prevalence as aging and obesity are escalating in developed populations. The knee is the joint most frequently affected by OA, characterized by cartilage deterioration and subchondral bone alterations, both tissues of mesenchymal origin, along with a high degree of synovial membrane inflammation, considered a hallmark of this disease [55,56,57].

Phase I clinical trials have demonstrated the safety of autologous or allogeneic intra-articular administration of MSCs in OA, showing a reduction of pain and inflammation and functional improvements [58,59,60,61,62,63]. Interestingly, when evaluating single autologous versus allogeneic MSC administration for OA, evidence from clinical trials point to the use of autologous MSCs as not being as efficient as allogeneic MSCs, probably due to the effects of aging in autologous MSCs. Thus, it has been reported that when using a single administration of autologous MSCs, higher doses of MSCs are needed to obtain beneficial outcomes (40–100 × 10^6^ cells) [61,63]. In contrast, a lower amount of allogeneic MSCs is reported to be able to obtain similar clinical improvements (25 × 10^6^ MSCs) [60,62]. Importantly, a randomized phase I/II clinical trial addressed the intra-articular injection of two repeated doses, six months apart, of 20 × 10^6^ allogeneic umbilical cord MSCs, along with a visco-supplementation treatment (hyaluronic acid), and demonstrated these as being safe and clinically superior to a single MSC dose [60]. These findings, together with the fact that single doses of MSCs have transient beneficial effects, likely as a result of low MSC retention and survival at the injection site, point to paracrine action of MSCs.

#### 3.1.2. Cardiovascular Disease

Cardiovascular diseases are among the most prevalent conditions in the elderly, encompassing almost half of all deaths in Europe [64]. The main rationale for using MSCs to treat cardiovascular diseases such as heart failure is based on their ability to exert paracrine effects that enhance cardiovascular regeneration and reduce fibrosis of scarred cardiac tissue [65,66,67]. Preclinical studies with MSCs administration in animal models of heart disease have also revealed improvements in the cardiac function of animals [68]. Accordingly, several phase I and II clinical trials have been conducted to treat heart failure, with encouraging results [69,70,71,72]. Thus, both autologous and allogeneic MSCs have been shown to be safe and to achieve clinical improvements in patients with cardiac disease. The mechanism of action points to MSCs’ secretory abilities. Improvements in quality of life and reductions in both major adverse cardiac effects and scar size have been detected in MSC-treated patients, attributed mainly to the paracrine action of MSCs [70,72]. Interestingly, a clinical trial comparing the efficacy of allogeneic versus autologous MSCs for non-ischemic dilated cardiomyopathy revealed increased improvements in the group of patients receiving allogeneic MSCs [73]. Thus, patients receiving a dose of 100 × 10^6^ allogeneic MSCs showed better outcomes in endothelial function and in the reduction of levels of the proinflammatory cytokine, tumor necrosis factor-alpha (TNF-α), usually increased in heart disease. Of note, greater clinical benefit has been obtained when administering a high dose of allogeneic MSCs (100 × 10^6^) to patients [72,74]. Again, the age of the MSC donors, which were in their 20s, was proposed as a major causal factor for the better outcomes in the group receiving allogeneic MSCs [73].

#### 3.1.3. Aging Frailty

Frailty, a nonspecific syndrome prevalent in advanced age populations, is characterized by reduced resilience leading to frail elderly individuals taking longer to recover after any type of stress (disease, traumatism), a situation strongly associated with adverse and serious outcomes. Women, who have a longer life expectancy, are more at risk for developing frailty [75,76]. Chronic inflammation and stem cell depletion are the key hallmarks of aging that most likely contribute to frailty [1]. Accordingly, MSC-based therapies have been proposed to ameliorate the signs and symptoms of this syndrome, thanks to their known immunomodulatory and paracrine actions [77]. Two successive Phase I/II trials (CRATUS study) have demonstrated the safety and efficacy of allogeneic bone marrow MSCs (age of the donors: 20–45 years in the Phase I trial and 19–27 years in the Phase II trial) for aging frailty [78,79]. In the Phase I clinical trial, the authors performed a nonrandomized MSCs dose-escalation study, and intravenously administered one infusion of 20, 100 or 200 × 10^6^ allogeneic MSCs (five subjects in each group; median age of 78.4 years). Outcomes were measured at 1, 3, 6 and 12 months post-infusion. In general, MSC therapy was safe and well-tolerated by the elderly. Moreover, there were improvements in some predictors of morbidity and mortality in aging frailty; all cell-dose groups showed increase scores in the 6 min walk distance, a validated test that assesses functional exercise capacity, three and six months post-infusion. Moreover, the group receiving 100 × 10^6^ cell-dose exhibited the most improvement in the physical component of the questionnaires on quality of life since the first month of infusion. Noticeably, patients receiving 100 and 200 × 10^6^ MSCs showed a significant decrease in serum levels of the proinflammatory cytokine TNF-α at six months post-infusion. Based on the outcomes of this study, the same authors performed a double-blind, placebo-controlled Phase II clinical trial, which consisted of a single dose of 100 or 200 × 10^6^ allogeneic MSCs (10 subjects in each group; mean age of 75.5 years). Of note, the outcomes of this latest phase II trial were quite similar to those observed in the Phase I trial, with the 100 × 10^6^ cells dose achieving the better outcomes. Although preliminary, these encouraging results deserve deeper examination to not only elucidate the underlying mechanisms of MSC therapy, but to delve into their efficacy, considering younger MSCs such as those from umbilical cord tissue, as ideal tools for treating aging frailty.

The hyperinflammation status triggered by COVID-19 disease has revealed the vulnerability of frail elderly individuals to this disease. In fact, frailty and older age have been proposed as determinants in the prognosis of COVID-19 disease, presenting these patients as being at an increased risk of severe clinical disease, with serious adverse and even lethal outcomes [80]. These facts that have been especially noticeable and dramatic in geriatric patients living in nursing homes [81]. MSC therapy was proposed at the very beginning of the pandemic in an attempt to find an effective treatment to fight the most severe cases of this emerging disease. In this unprecedented situation, the rationale for using MSCs as an efficient therapy was based on their known immunomodulatory and anti-inflammatory properties, as well as their tropism for lung tissue, where they are retained due to hemodynamic reasons [82,83]. Moreover, molecular studies reported that MSCs barely express ACE2 and TMPRSS2, two key plasmatic membrane proteins for SARS-CoV-2 entry, and therefore were free from infection upon transplantation in COVID-19 patients [84,85]. Both open-label, non-controlled, as well as randomized double-blind phase 1/2a trials, using MSCs from different sources, revealed no serious adverse events, and positive results in decreasing inflammatory parameters and improving patients’ survival and time to recovery [84,86,87]. While phase III trials are needed to further characterize the MSCs’ mechanisms of action, and outcomes in diminishing mortality and lung effects in COVID-19 patients, it is worth to mention that in April of 2020, the United States Food and Drug Administration approved MSC treatments for “expanded access compassionate use” for seriously ill COVID-19 patients.

### 3.2. MSCs to Counteract Premature Aging Syndromes

As previously mentioned, HGPS, also known as progeria, is a devastating progeroid laminopathy, with no current curative treatment. Although never carried out, the possibility of administering MSC therapy to treat HGPS patients that show MSC functional impairment as mentioned above, has long been considered in light of the encouraging regenerative and paracrine properties of these cells. The immunomodulatory properties of MSCs could also justify using them as a therapy to counteract premature aging syndromes. Thus, an excess of proinflammatory signals, such as high levels of IL-6 expression and the activation of the NLRP3 inflammasome, have been described in both human HGPS cells and in progeria animal models [54,88]. Moreover, the specific inhibition of these mediators of inflammation ameliorate progeroid features and improved lifespans in progeroid mice models point to a causal relationship between progerin expression, inflammation and premature aging [54,88]. Interestingly, a recent case report addressed the potential benefits of a cell therapy based on allogeneic haploidentical adipose stromal vascular fraction (SVF) for HGPS, with quite encouraging results [89]. In this study, the authors isolated and administered two infusions (one week apart) of SVF isolated from the patient’s mother and composed of a heterogeneous cell population (including MSCs) to a 12.8-year-old HGPS patient. It is important to mention that this patient was previously (during ≈ 7 years) under lonafarnib treatment, but this medication was discontinued due to side effects. The patient’s weight and height as well as serum markers were analyzed before and after the treatment (0.5, 1, 2 and 4 months). Among the markers analyzed, the authors only found, two months after the treatment, a rise in the serum levels of insulin-like growth factor 1 (IGF-1), a molecule which peaks in puberty and correlates with height gain [90]. Interestingly, decreased IGF-1 levels have been shown in progeroid mice accumulating prelamin A, in which supplementation with this factor was able to ameliorate premature aging features [91]. Consistently, with these findings, the body height and weight of the HGPS pediatric patient also showed an appreciable increase four months after the therapy (5 cm and +1.2 kg respectively), which are considerably higher than those showed by HGPS patients per year (an average of 3.5 cm and 0.5 kg respectively). Considering the observed clinical benefits, even when using cell therapy coming from a non-young donor, this positive result opens new avenues in treatments for HGPS patients by exploring the clinical potential of allogeneic young MSCs or their derivatives to treat this devastating disease.

## 4. MSC-Based Cell-Free Therapy for Aging

Emerging data from bench, basic studies to preclinical and clinical evidence, support the idea that the mechanism of action of MSCs administered to human patients and animal models mainly lies in their ability to secrete bio-active factors [92]. Thus, via paracrine action, MSCs exert short or long intercellular communication by releasing soluble mediators (growth factors and cytokines) and membrane-based EVs. The latter can be sorted by size in microvesicles (100–1000 µm) and exosomes (40–200 µm), both of them containing high concentrations of mRNA, miRNA, proteins and lipids.

As mentioned above, heterochronic parabiosis experiments showed that healthy blood components (young or exercised), may benefit age-related deficits, thus pointing to specific factors, such as EVs, functioning as intercellular messengers to counteract aging consequences. Supporting this observation, EVs produced by different cell types, such as fibroblasts and stem cells, ameliorate physiological and premature age-related deterioration when tested, in both in vitro and in murine models of aging [93,94,95,96]. Interestingly, EV production by stem cells increases depending on the stemness of the cells. Thus, iPSCs have been shown to produce significantly more EVs than neonatal or adult MSCs, and in turn, neonatal MSCs produce more EVs than adult MSCs do [96]. Moreover, the internalization efficiency of EVs derived from iPSCs in target cells is higher than that of EVs derived from tissue MSCs [96]. Consistently, EVs coming from young MSCs show superior functional abilities, mainly attributed to their different cargo. Thus, EVs derived from young MSCs exhibit superior immunomodulatory ability when compared to EVs coming from MSCs isolated from older donors, mainly due to different levels of specific miRNAs in the young EVs [97]. The high concentration of antioxidant enzymes in EVs isolated from fibroblasts and MSCs has also been pointed to as a mechanism for overcoming oxidative stress of senescent cells treated with EVs [95,96].

In view of these outcomes, it is clear that the source origin of MSCs and the inherent age of that source also define the properties of secreted EVs. Thus, EVs coming from neonatal MSCs (isolated from umbilical cords) are also richer in anti-aging signals than the EVs derived from adult MSCs [93,97]. Treatment of primary elderly human or HGPS fibroblasts with EVs derived from young, but not old, primary fibroblasts, reduced classical in vitro markers of senescence in these cells, such as β-galactosidase staining and impaired cell proliferation. Injection of these young EVs also reduced senescence markers in a number of tissues of elderly mice [95]. This is consistent with a recent study comparing blood EV features from different age donors. Thus, whereas blood EV size, concentration and total protein content remains constant across young, middle-aged and elderly subjects, the EVs’ protein composition did change with age. Interestingly, in silico analysis of EV proteomics suggest that this age-dependent difference in protein cargo could probably be due to different activities of certain cell types over time, with circulating EVs mainly coming from blood cells, bone marrow and the pancreas in aged individuals [94]. Considering this evidence, intense research efforts are being addressed to develop effective cell-free therapies in the context of aging and age-related diseases, based on EVs derived from young stem cells, especially umbilical cord-derived MSCs (UCEVs) and iPSCs. Thus, the striking findings reported by two recent works based on UC-EV treatment in aged mice models point to the fact that a therapy based on UC-EVs to counteract human aged-related diseases is a step closer to being established [93,98]. Lei and coworkers showed that UC-EVs contain more anti-aging signals, in particular transcripts for genes related to cell cycle and DNA replication and repair, than EVs derived from adult human bone marrow MSCs (>40 years donors). Consistently, UC-EVs were shown to partially rejuvenate adult MSCs by transferring mRNA transcripts of proliferating cell nuclear antigen (PCNA). These rejuvenated adult MSCs showed superior regenerative capacities both in vitro and in vivo (animal models) in terms of osteogenic differentiation, wound repair and angiogenesis [93]. Finally, the authors studied the short-term effects of UC-EV therapy by intravenously injecting UC-EVs in elderly mice during four weeks. Interestingly, the production of proinflammatory cytokines in elderly mice was diminished after UC-EV treatment, and regeneration in tissues affected by age, such as bone and kidney, was also observed. Almost simultaneously, another work showed similar in vitro results regarding the potential of young bone marrow MSCs-EVs to rejuvenate senescent BM-MSCs. Moreover, the authors showed that just two injections of EVs derived from young MSCs were able to extended the life and healthspan in both natural and progeroid mouse models of aging [98].

Also noteworthy is a recent non-randomized clinical trial exploring the safety and efficacy of exosomes derived from allogeneic bone marrow MSCs to treat severe COVID-19 patients showing moderate to severe acute respiratory distress syndrome (ExoFlo^TM^) [99]. Remarkably, a single intravenous dose of exosomes was demonstrated as safe and improved clinical parameters such as oxygenation and inflammatory markers, especially within the first three to four days after EV infusion.

Collectively, this evidences points to young EVs derived from MSCs as being a key tool for counteracting both systemic aging and age-related pathologies, as a result of their healthy or youthful cargo. In fact, a cell-free therapy based on EVs seems to be more appealing than a cell therapy: it could be a well-characterized off-the shelf product without the safety concerns of using a “living” product such as MSCs. However, before EV-based therapies enter the clinic, further basic and preclinical research is needed in order to obtain a well-defined clinical product. Knowledge about specific aspects of EV biology, such as appropriate EV characterization, the specific molecules driving the rejuvenation process and the cell populations’ target of EVs, will be fundamental to unravelling the underlying mechanisms of EV therapy in the aging process.

## 5. Harnessing the Efficacy of MSC-Based Therapies

A striking feature of MSCs is that they show dynamic paracrine profiles and functional capabilities, depending on the environment they face [100]. This ability of MSCs to mount an adaptive response is being explored in vitro to enhance their therapeutic potential, by mimicking the microenvironment the MSCs are going to face in vivo. For this purpose, the cell culture conditions are modulated in a process known as “preconditioning”, “licensing” or “priming”. To date, the most-studied priming strategies enhancing MSC properties are hypoxia, resembling the low oxygen concentrations of the disease site, and the stimulation of MSCs with molecules that are abundant in the target injured site, such as pro-inflammatory cytokines in immune diseases, or even the bacterial wall component LPS in skin wounds [100,101,102]. Thus, the immunosuppressive capacity of MSCs is enhanced by exposure to the proinflammatory cytokine interferon gamma (IFN-γ), even in the case of senescent MSCs, and by the up-regulation of the proteins indoleamine 2,3-dioxygenase (IDO) and programmed cell death ligand 1 (PD-L1) [103,104,105]. These priming strategies are currently going a step forward in an attempt to mimic more closely the disease microenvironment milieu that MSCs are going to encounter upon administration. Thus, the in vitro stimulation of MSCs with disease-specific plasma, such as stroke or graft versus host disease, has also been proposed as an effective priming strategy [105,106]. In our opinion, MSC priming strategies will be of paramount importance for enhancing the clinical potential of these cells or their derivatives in age-related pathologies, a currently unexplored option. Further research in this exciting area of knowledge will shed light on the mechanisms driving in vitro and in vivo improvements by cell priming approaches.

## 6. Future Directions and Conclusions

The personal, social and economic burden of age-related pathologies have long been an increasing public health concern, a fact dramatically revealed with the current COVID-19 pandemic especially affecting the elderly. In this context, MSC-based (cell and cell-free) therapies emerge as quite a compelling option to counteract aging, one that deserves further research. By 2020, ten MSC products have achieved regulatory approval to treat mainly graft versus host disease (GVHD), myocardial infarct, neurological disorders, Chron’s fistula and knee articular defects, the latter being the only one using umbilical- cord-derived MSCs [9]. This encouraging fact reflects the fact that after decades of basic and clinical research, the inherent drawbacks of using a living product (multiple levels of heterogeneity, dose and administration route, among others) can be surmountable.

**Figure 1:**
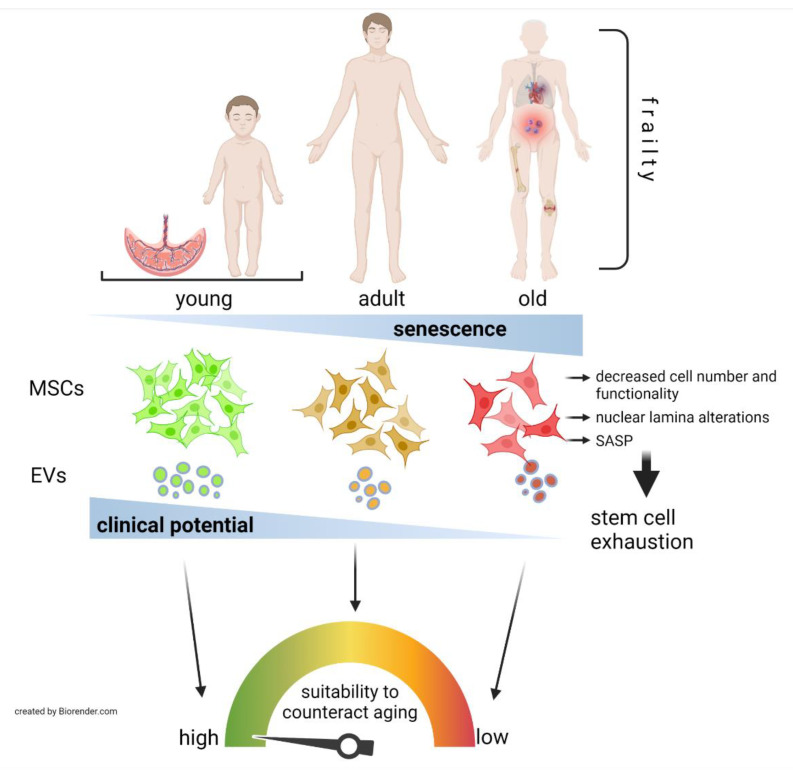
Age-related suitability of MSCs or derived products to be used as therapeutics to counteract human aging or age-related pathologies. MSCs’ number and functional capabilities diminish as the donor’s age increases, with those isolated from neonatal tissues, or their EVs, such as umbilical cord MSCs showing the greatest clinical potential. Thus, elderly individuals affected by systemic conditions such as inflammation and frailty or by age-related pathologies such as cardiovascular disease, acute respiratory distress syndrome (shown in severe COVID-19 patients) and musculoskeletal conditions (fractures, OA) among others, would be especially benefitted by young MSC-based treatments.

As highlighted in this review, when intended for counteracting aging and age-related pathologies, the youthfulness of clinical-grade MSCs, reflected by the age of MSC donors and the number of passages in culture, should be a major point to consider before addressing the therapy, since it will be fundamental in determining the therapeutic potential of MSCs (Figure 1). This observation is supported by recent studies comparing the efficacy of MSCs from different sources in preventing inflammatory diseases such as GVHD in murine models [107].

Not only the youthfulness of MSCs seem to be essential for the success of the therapy—host factors seem also to be decisive. Thus, the disease stage/severity of each patient, such as immune status, hypoxia, inflammation or extracellular matrix alterations, can affect the therapeutic outcomes of MSCs [9]. Along these lines, our recent clinical trial assessing repetitive HLA-matched young MSC infusions in two pediatric patients affected by the rare bone disorder osteogenesis imperfecta reported better beneficial outcomes, and systemic pro-osteogenic response in the most severely affected patient [13].

All in all, the evidences summarized in this review suggests that cell-based or cell-free therapies based on young MSCs should be considered as realistic interventions to counteract aging. Moreover, the clinical benefits of MSCs could be enhanced by in vitro cell priming and then administered concomitantly with other drugs, to enhance the final therapeutic outcomes. These currently unexplored approaches will undoubtedly deserve and require more research, to bring a new era of advanced therapeutics for improving the healthspan of individuals by preventing or delaying many of the pathologies of aging.

## Data Availability

Not applicable.
